# BERTwalk for integrating gene networks to predict gene- to pathway-level properties

**DOI:** 10.1093/bioadv/vbad086

**Published:** 2023-07-03

**Authors:** Rami Nasser, Roded Sharan

**Affiliations:** School of Computer Science, Tel Aviv University, Tel Aviv 69978, Israel; School of Computer Science, Tel Aviv University, Tel Aviv 69978, Israel

## Abstract

**Motivation:**

Graph representation learning is a fundamental problem in the field of data science with applications to integrative analysis of biological networks. Previous work in this domain was mostly limited to shallow representation techniques. A recent deep representation technique, BIONIC, has achieved state-of-the-art results in a variety of tasks but used arbitrarily defined components.

**Results:**

Here, we present BERTwalk, an unsupervised learning scheme that combines the BERT masked language model with a network propagation regularization for graph representation learning. The transformation from networks to texts allows our method to naturally integrate different networks and provide features that inform not only nodes or edges but also pathway-level properties. We show that our BERTwalk model outperforms BIONIC, as well as four other recent methods, on two comprehensive benchmarks in yeast and human. We further show that our model can be utilized to infer functional pathways and their effects.

**Availability and implementation:**

Code and data are available at https://github.com/raminass/BERTwalk.

**Contact:**

roded@tauex.tau.ac.il

## 1 Introduction

Deep learning techniques are transforming the field of data science and have become the state of the art in a range of applications in biology and medicine (Ching *et al.*, 2018). A graph is a common and general data structure to express relations between elements. Various shallow and deep learning strategies were proposed for graph representation learning. These include shallow or deep encoder–decoder schemes (Grover and Leskovec, 2016; Kipf and Welling, 2016) to learn a latent embedding of the nodes, which minimizes a graph reconstruction loss, graph neural networks (GNNs) (Zhou *et al.*, 2020) to relate between features of connected entities, and hybrid methods that use GNNs for the encoding.

Recently, such a hybrid technique called BIONIC was applied to analyze and integrate biological networks (Forster *et al.*, 2022), demonstrating state-of-the-art results in comparison to the iCell (Malod-Dognin *et al.*, 2019) matrix factorization approach and shallow learning approaches, such as mashup (Cho *et al.*, 2016) and node2vec (Grover and Leskovec, 2016), in co-annotation prediction, module detection and gene function prediction. Nevertheless, its encoder, decoder and integration components were arbitrarily defined and suffered from several shortcomings. Specifically, encoding started from an arbitrary initialization of each node’s feature vector, and cross-learning between nodes was performed by neighborhood averaging using graph attention layers which limits learning to nodes that are as far as the number of layers used (2 in this case). Decoding was arbitrarily defined based on inner product and as the input networks tend to be sparse, this leads to high dimensional embedding. For integration, each network uses its own encoding layers and the final embedding is arbitrarily averaged over all networks.

To address these shortcomings, we developed a novel deep learning method that tackles the graph representation learning challenge by transforming the graph structure into a text-like structure using random walks and employing a state-of-the-art masked language model (BERT; Devlin *et al.*, 2019) to achieve the encoding. Importantly, the data transformation allows seamless integration of networks by collecting random walk information from all networks and learning a joint embedding. The transfer of information from distant nodes is achieved via the random walks and does not require any additional parameters to learn. Propagation steps during training smooth the embedding learned across the networks.

We apply our model, which we call BERTwalk, to two comprehensive benchmarks of yeast and human networks and show that BERTwalk outperforms BIONIC and four other previous methods in a range of clustering and classification tasks. Importantly, Our BERTwalk model is trained using a masked language modeling strategy, enabling its use as a pre-trained model for pathway-level downstream tasks that involve a sequence of genes as input (rather than single genes or gene pairs). We exploit this property to successfully infer functional pathways and their effects.

## 2 Methods

We introduce a new model, which we call BERTwalk, for unsupervised learning of a graph structure for downstream tasks. Let G=(V,E,w) be a graph on a set *V* of nodes and a set *E* of edges whose weights are given by a function w:E→R. Let *A* be the graph’s adjacency matrix such that $A_{ij}=w(i,j)$ for any (i,j)∈E, and $A_{ij}=0$ otherwise. Finally, let $D_{ii}=\sum_{j}A_{ij}$ be the corresponding diagonal degree matrix.

**Figure 1.**
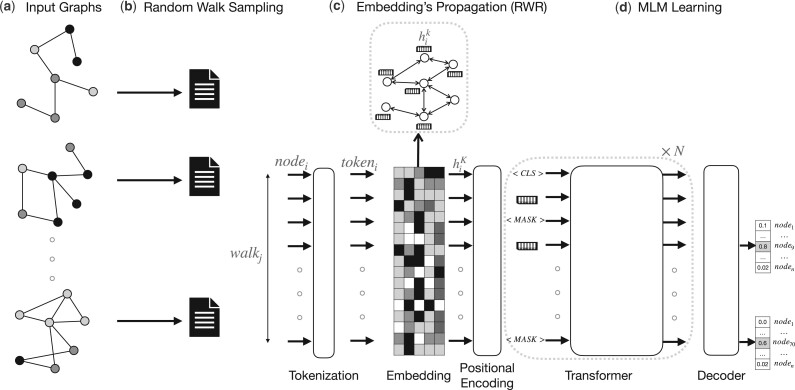
A sketch of the proposed BERTwalk model. (**a** and **b**) Networks are fed into the model and every network is transformed into a collection of node sequences, produced via random walks, and a special symbol [CLS] is added at the beginning of every sequence. (**c**) The resulting corpus is fed to a transformer model, standard pre-model steps take place in order to numerically represent the data (tokenization and embedding), embedding is propagated every epoch on one of the input networks (iterating over them) before it is fed to the transformer which learns a vector representation for each input that can be used to predict the class of a walk (sequence of nodes) or the identity of masked nodes. (**d**) The model is trained by masking part of the nodes and training the model to predict them. The end result is an embedding of the nodes, taken from the Embedding layer

BERTwalk receives as input a collection of *T* networks $\left\{G_{1},G_{2},\ldots G_{T}\right\}$ on the same set of *n* nodes, and a number *e* of desired embedding dimension; the goal is to learn a node embedding matrix $X\in R^{n\times e}$. BERTwalk includes several parts: (i) random walk sampling, (ii) node embedding propagation, (iii) graph-transformer-based encoder, (iv) masked language task learning and (v) node feature representation. A high level description of the algorithm is given in Figure 1. The different steps are described in detail below.

### 2.1 Random walk sampling and embedding via propagation

To capture network structural information and to convert it to a text-like structure, we encode paths in the network as sentences using random walks as in Grover and Leskovec (2016). The walk transitions from a node to one of its neighbors with probability proportional to the weight of the edge connecting them. Similarly to Grover and Leskovec (2016), we perform random walks of length 10, repeating 10 times for every node in every input network. To ensure robustness of our results to the length of the walk used, we tested different lengths ranging between 10 and 80 and evaluated the performance of BERTwalk on each of the three yeast integration tasks described below (Section 3). We observed that the results were robust to the length used with all differences insignificant (ANOVA p>0.91).

To encode these walks, the model initiates an embedding matrix $x\in R^{n\times e}$ with *n* the size of the vocabulary (number of nodes) and *e* is the desired embedding’s dimensionality. While the input networks we consider are undirected, the walks are directed, hence there is a need for incorporating the order of the nodes into our model. A common way to achieve that is by positional encoding of the nodes, where we use the following scheme (Ashish *et al.*, 2017):


(1) $$PE_{\left(\mathrm{pos},i\right)}=\{\begin{array}{cc} \;\text{sin}(\frac{\mathrm{pos}}{10000^{i/e}}) & \;\text{if}\;i\;\text{mod}\;2=0\\ \;\text{cos}(\frac{\mathrm{pos}}{10000^{(i-1)/e}}) & \;\text{otherwise} \end{array}.$$

$PE_{\left(\mathrm{pos},i\right)}$ represents the *i*th coordinate of the position encoding at position *pos* in the sequence. These values are concatenated to the original input features (embedding matrix).

**Figure 2.**
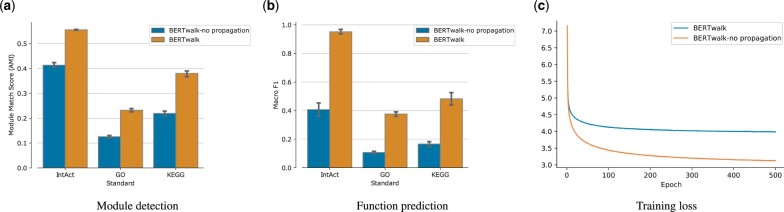
Effect of propagation during training. (**a** and **b**) The propagation procedure enhances performance in downstream tasks. (**c**) Cross-entropy training loss as a function of the epoch for the masked language modeling task

The embedding matrix and the decoder layer are initialized uniformly at random. For the transformer layer, We utilized Xavier’s initialization method (Devlin *et al.*, 2019). These parameters are updated by the model during training. The first token of every sequence is always the classification token ([CLS]), and as other tokens, it has corresponding vector in embedding matrix. The final hidden state of the first token of a particular sequence is used as sequence representation (aggregator) for sequence level tasks such as classification. To further smooth the learned vectors according to the graph structure, we propagate them every epoch of training using one iteration of random walk with restart: where $W=D^{-1/2}AD^{-1/2}$ and $X^{\prime }$ is the propagated embedding used in the epoch. The propagation procedure at every epoch can be considered as a way of regularization that ensures smoothness of the embedding over the network. Indeed, while the loss function is lower when not using the propagation (Fig. 2c), the learned features are more informative for downstream tasks (Fig. 2a and b).


$$X^{\prime }=(1-\alpha )WX+\alpha X,$$

### 2.2 Masked language modeling

Since the input nodes of the networks are unlabeled, we use a masked language modeling (MLM) approach for training as used by BERT (Devlin *et al.*, 2019). The main model component is a transformer encoder which receives a sequence of vectors (representing tokens) and transforms them by applying a sequence of N=4 identical blocks. Each block consists of a self-attention mechanism followed by feedforward neural network, as detailed in Ashish *et al.* (2017). For completeness, we describe the network structure briefly here. Let $H=[h_{1},h_{2},\ldots ,h_{L}]$ be an input sequence of *L* tokens, where each token is represented by a *d*-dimensional vector. A self-attention layer applies the following transformation: where $Q=W_{q}H,K=W_{k}H$ and $V=W_{v}H$ for learnable weight matrices $W_{q}$, $W_{k}$ and $W_{v}$. The feed-forward layer further is applied to each position separately and identically. This consists of two linear transformations with a ReLU activation in between, transforms the result as follows: for learnable matrices $W_{1},W_{2}$ and vectors $b_{1},b_{2}$.


$$\begin{array}{c} \mathrm{Attention}(Q,K,V)=\mathrm{softmax}\left(\frac{QK^{T}}{\sqrt{d_{k}}}\right)V \end{array},$$

$$\text{FFN}(x)=\mathrm{ReLU}(xW_{1}+b_{1})W_{2}+b_{2},$$

The MLM task in BERT involves randomly masking some of the input tokens and then predicting the masked tokens based on the remaining context. This task encourages the model to learn contextual relationships between words and representations that capture information from both left and right context. Specifically, we mask 20% of the tokens (representing nodes) in each generated sentence and aim to identify the masked token identity using cross-entropy as a loss function: where *B* is the batch size, *L* is the sequence length, *M* is the number of classes, *y* a binary indicator which is 1 if node label *c* is the correct classification for observation (b,l), and *p* is the predicted probability of observation (b,l) to be labeled *c*.


(2) $$L=-\sum_{b=1}^{B}\sum_{l=1}^{L}\sum_{c=1}^{M}1_{\left\{b,l\in \mathrm{mask}\right\}}\cdot y_{b,l,c}\;\text{log}\;p_{b,l,c},$$

### 2.3 Data description and performance evaluation

In this study, we apply our model to six networks, including three yeast networks and three human networks. The yeast networks consist of correlated genetic interaction profiles network of Costanzo *et al.* (2016), a co-expression network derived from transcript profiles of yeast strains carrying deletions of transcription factors of Hu *et al.* (2007) and a protein–protein interaction network obtained from an affinity-purification mass-spectrometry assay of Krogan *et al.* (2006). The human data, taken from Malod-Dognin *et al.* (2019), include a protein–protein interaction network from IID database v.2016-03 (Kotlyar *et al.*, 2016), a co-expression network from COXPRESdb v.6.0 (Okamura *et al.*, 2015) and genetic interactions from BioGRID v.3.4.137 (Chatr-Aryamontri *et al.*, 2017). Overall, the yeast networks that we analyze here result in 5232 unique nodes and a corpus of 52 320 sentences (walks) from which the embedding is derived, and the human networks span 10 080 unique nodes and a corpus of 100 800 sentences (walks). The number of nodes and edges in each network are provided in Table 1.

**Table 1.** Statistics of the input networks

Network	Organism	No. of nodes	No. of edges
Costanzo *et al.* (2016)	Yeast	4529	33 056
Hu *et al.* (2007)	Yeast	1101	14 826
Krogan *et al.* (2006)	Yeast	2674	7075
Kotlyar *et al.* (2016)	Human	13 310	164 152
Okamura *et al.* (2015)	Human	13 063	156 820
Chatr-Aryamontri *et al.* (2017)	Human	3111	10 266

In order to evaluate the computed embedding, we use the recent benchmark of BIONIC (Forster *et al.*, 2022), which focuses on three tasks: (i) gene co-annotation prediction; (ii) gene module detection; and (iii) supervised gene function prediction. As tasks (i) and (ii) are evaluated based on the same categorization and due to the essentially identical goal of tasks (i) and (iii), we focused here on (ii) and (iii) only. Functional benchmarks were derived from IntAct protein complexes (Orchard *et al.*, 2014), Kyoto Encyclopedia of Genes and Genomes (KEGG) pathways (Kanehisa and Goto, 2000) and Gene Ontology biological processes (GO) (Ashburner *et al.*, 2000).

### 2.4 Implementation and runtime details

Our model was implemented in PyTorch. For training the model we used Adam optimizer to update the weights of the network and the embedding. BERTwalk average epoch time when applied to the yeast dataset described above was 1.2 min (compared to 1.5 min for BIONIC) when executed on NVIDIA TITAN GPU with 12 GB RAM. During training, we used batch size of 64 sequences, which allows scalable training regardless of the number and size of the networks. We learned node embeddings of size e=128.

The embeddings for all other integration methods were taken from the BIONIC publication (Forster *et al.*, 2022), where implementation details of all methods are available. The embedding size for each of these previous methods is 512.

## 3 Results

### 3.1 Overview

We developed a transformer-based model for network integration and representation learning. Our framework transforms each input network into text-like sentences via random walks; these sentences are concatenated across networks and the resulting corpus is fed to a BERT transformer to embed nodes for downstream clustering and classification tasks. Unique to our approach is the use of network propagation steps during learning to ensure smoothness over the network of the learned embeddings. We benchmark BERTwalk using a recent yeast network benchmark and compare to state-of-the-art previous methods including BIONIC (Forster *et al.*, 2022), iCell (Malod-Dognin *et al.*, 2019), a deep learning multi-modal autoencoder deepNF (Gligorijević *et al.*, 2018), Mashup (Cho *et al.*, 2016) and a multi-network extension of the node2vec model called multi-node2vec (Wilson *et al.*, 2021). We further assess the performance of our method on a human benchmark.

### 3.2 A yeast benchmark

The first task in which we evaluate our approach is an unsupervised task in which node embeddings are used to identify gene modules. The results are evaluated based on several annotated collections of protein complexes, pathways and biological processes taken from IntAct, KEGG and GO, respectively. The evaluation was done following Forster *et al.* (2022) by hierarchically clustering the learned embedding using a variety of distance metrics, linkage methods and thresholds and comparing the coherency of the resulting clusters with the module-based standards according to an adjusted mutual information (AMI) measure. AMI measures the similarity between two clusterings while accounting for chance agreement (see Forster *et al.*, 2022 for full details).

**Figure 3.**
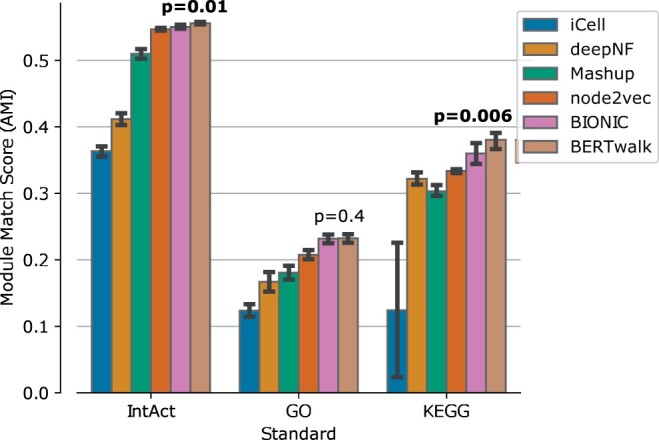
Module detection performance on yeast benchmark measured using the adjusted mutual information (AMI) measure. *T*-test *P*-values for performance differences between BERTwalk and the second-best method (BIONIC) appear above the bars

Notably, a similarity measure between nodes used in this task is cosine similarity which involves dot-product of their feature vectors. Dot-products are the decoding operations used by BIONIC and the algorithm is optimized using the difference between those dot-products and the input gene networks. As the input networks are correlated with module co-membership, the results may be biased in favor of BIONIC’s training approach. Nevertheless, as shown in Figure 3, BERTwalk compares favorably to BIONIC and to other previous methods across all three collections.

**Figure 4.**
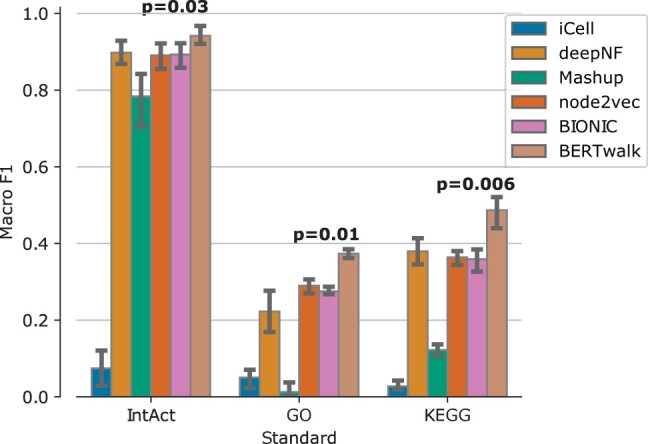
Gene function prediction performance on yeast benchmark evaluated by Macro F1 which is the average per-class F1 score (harmonic mean of precision and recall). *T*-test *P*-values for performance differences between BERTwalk and the second-best method appear above the bars

The second task is a supervised one, where the goal is to use node embeddings to predict gene function. We used IntAct, KEGG and GO gene function prediction standards, where terms with less than 10% frequency were filtered out to allow proper stratification when splitting the data to 5-folds during cross-validation. The task here is multi-label classification where each protein can have multiple, potentially dependent labels. Thus, we used a random forest classifier which is suited to this kind of tasks and can handle label dependency. For each method, we used 5-fold cross-validation to evaluate its embedding performance, measured by the average over the 5-folds of the macro F1 score which calculates the F1 score for each label separately and then takes the average of these scores. The results are summarized in Figure 4 and show that BERTwalk outperforms all other methods across the three standards.

**Figure 5.**
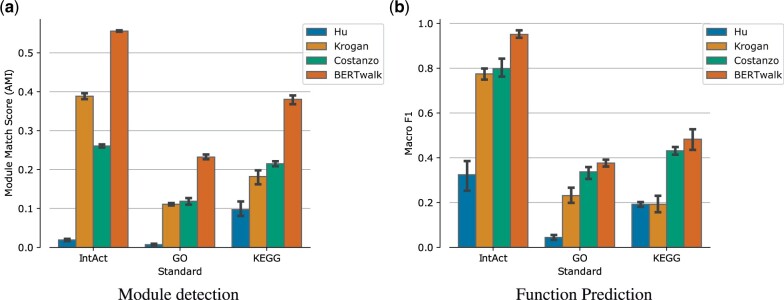
Comparison of BERTwalk integration to analyzing each network separately

Importantly, when comparing the full integrative pipeline to operating on each network separately, we observe the power of data integration with superior performance in all tested scenarios (Fig. 5).

### 3.3 Integration of human networks

**Figure 6.**
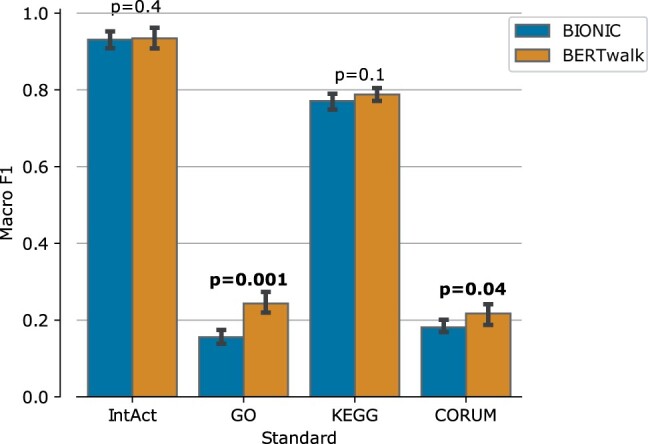
Human gene function prediction performance evaluated by Macro F1 which is the average per-class F1 score (harmonic mean of precision and recall). *T*-test *P*-values for performance differences between BERTwalk and BIONIC appear above the bars

After establishing the accuracy of our framework on the yeast benchmark, we turned to examine its performance on an independent benchmark of network integration in humans. We integrated three human molecular interaction networks as detailed in Table 1 and learned a global embedding for each protein using BERTwalk. In order to evaluate the learned embeddings, we followed the yeast evaluation and built an analogous benchmark of functional annotations using the GO, KEGG and IntAct resources as well as the CORUM collection of protein complexes (Giurgiu *et al.*, 2019). For comparison purposes, we trained BIONIC with their recommended parameter setting for humans. As in the yeast case, the node embeddings were fed to a random forest classifier to predict the functional class. The results are summarized in Figure 6 and show that BERTwalk outperforms BIONIC across the four annotation standards.

### 3.4 Supervised pathway prediction

**Figure 7.**
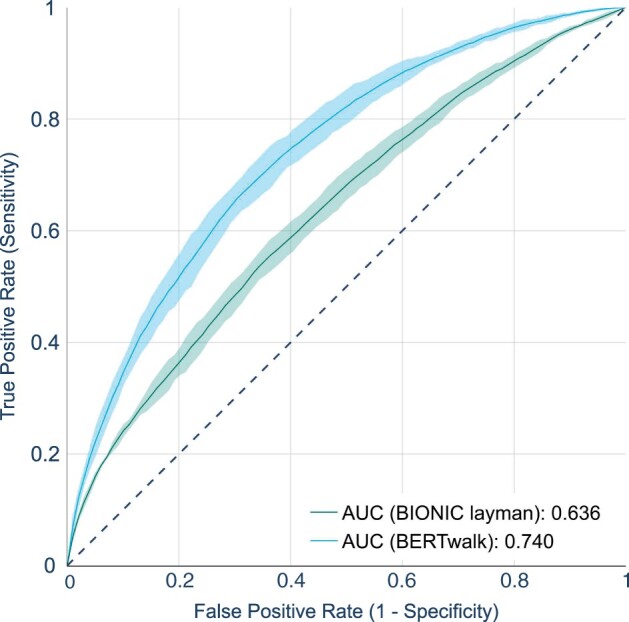
Pathway prediction performance. Pathway representations were derived from BERTwalk and BIONIC layman and used to train a classifier of pathway category (–1/0/1). Shown are the resulting macro-averaged one-class versus rest ROC curves

One advantage of BERT-like models is the sentence representation they learn in the form of the [CLS] token embedding that can be used for downstream tasks, such as sentiment analysis in NLP. Here, we demonstrate the power of using this representation in the biological domain for pathway learning. We use a broad definition of a pathway as a chain of interacting proteins leading from a mutated protein to an affected protein. Specifically, we synthesize protein pathways using the PPI network of Krogan *et al.* (2006) and knockout gene expression data from Kemmeren *et al.* (2014). We consider as pathways those paths that start with a deleted mutant and end in a differentially expressed gene (Yeang *et al.*, 2004). Such a pathway is labeled 1 if the gene is upregulated and −1 if it is downregulated. All other paths are labeled 0. In total, we constructed 26 740 paths, 18% of which are nonzero. The input path is represented as single sequence, and the final hidden vector corresponding to the first token ([CLS]) serves as an input to a multi-label (1/0/−1) classifier. For classification, we used random forest which we train using the pathway data in a cross-validation setting. For comparison purpose, we also devised a layman approach in which BIONIC node features are averaged over the path and the resulting vector serves as input for the classifier. The classification results in a 5-fold cross-validation setting are depicted in Figure 7, with BERTwalk outperforming the layman approach and attaining an area of 74% under the curve, computed by macro-averaging one-class versus rest ROC curves.

In order to assess the biological relevance of the synthesized paths from knockout to an effect, we compared the functional enrichment of 0-labeled paths against paths that are labeled 1 or −1. For each path, we computed a hypergeometric *P*-value for its enrichment with a GO term (taking the minimum over all terms). Subsequently, we performed a Wilcoxon rank sum test to compare the obtained *P*-values between the two path categories. Our analysis revealed a significantly higher enrichment of the ±1-labeled paths (*P* < 0.043).

## 4 Conclusions

We have developed a novel deep learning framework, BERTwalk, for integrative analysis of biological networks. Our framework transforms network data into text-like documents via random walks and uses the BERT transformer to embed nodes for downstream clustering and classification tasks. We show the superiority of our method over the state-of-the-art BIONIC encoder–decoder scheme using both yeast and human benchmarks. We further demonstrate its utility in predicting pathway-level properties. The ability to embed whole pathways could be potentially used to discover novel signaling pathways and annotate their functional properties.
